# Functional Analysis of *MysERG1*, a Novel Immune-Related Gene in Encapsulation Regulation, in the Oriental Armyworm *Mythimna separata* (Lepidoptera: Noctuidae)

**DOI:** 10.3390/insects17040372

**Published:** 2026-04-01

**Authors:** Puyuan Guo, Seiichi Furukawa

**Affiliations:** 1 Degree Programs in Life and Earth Sciences, University of Tsukuba, Tsukuba 305-8572, Ibaraki, Japan; 2 Institute of Life and Environmental Sciences, University of Tsukuba, Tsukuba 305-8572, Ibaraki, Japan

**Keywords:** cellular immunity, hemocytes, plasmatocytes, granulocytes

## Abstract

Encapsulation is a major cellular defense reaction in lepidopterans. However, in the oriental armyworm (*Mythimna separata*), the molecular regulators that coordinate hemocyte adhesion and multilayer capsule assembly remain poorly defined. In this study, we identified *Mythimna separata encapsulation related gene 1* (*MysERG1*) as a novel cellular immune regulatory gene. *MysERG1* transcripts were most abundant in hemocytes and were notably upregulated in adherent hemocytes as well as in samples of capsules, indicating an association with adhesion-dependent hemocyte activation. Following separation of granulocytes and plasmatocytes, *MysERG1* expression was observed to be higher in adherent plasmatocytes than in adherent granulocytes. However, recombinant MysERG1 selectively increased granulocyte adhesion but did not significantly affect plasmatocyte adhesion and was specifically localized on granulocytes. Additionally, recombinant MysERG1 enhanced hemocyte aggregation on foreign surfaces, highlighting its functional role in facilitating encapsulation. Functional knockdown using double-stranded RNA significantly reduced the size of the capsules, indicating that MysERG1 is required for robust capsule formation. This study identifies MysERG1 as a novel factor involved in hemocyte cooperation during encapsulation in *M. separata* and presents a conceptual framework for inter-hemocyte communication mechanisms in lepidopteran cellular immunity.

## Simple Summary

Insects possess an innate immune system to protect themselves from the threat of various intruding microorganisms. Encapsulation is one of the cellular immune reactions against large invaders such as parasitoid eggs, and a large number of hemocytes surround and isolate them in a multilayered capsule. Encapsulation capsules are composed of two adhesive hemocyte types: granulocytes and plasmatocytes. However, the signals that coordinate different hemocyte types’ attachment to each other and build the multilayered capsules are not fully understood. In this study, we identified a previously unidentified gene in the oriental armyworm, *Mythimna separata*, and named it *MysERG1*. *MysERG1* was highly expressed in hemocytes that attach to foreign surfaces and participate in capsule formation. Although *MysERG1* was highly expressed in adherent plasmatocytes, the recombinant protein was localized in granulocytes. When *MysERG1* expression was suppressed using RNA interference, both hemocyte density and capsule size were reduced, indicating that *MysERG1* is required for proper capsule development. These results suggest that MysERG1 of *M. separata* functions as a novel factor that mediates communication between granulocytes and plasmatocytes during the encapsulation process. *MysERG1*-like sequences were conserved in lepidopteran insects specifically, implying that MysERG1 plays an important role in immunity in Lepidoptera.

## 1. Introduction

The innate immune system of insects is critical in the defense against pathogen invasion and parasitoid infections. In contrast to vertebrates, insects lack adaptive immunity, and their defense relies primarily on innate humoral and cellular immune responses [1]. Cellular immunity is characterized by reactions such as phagocytosis, nodulation, and encapsulation, which are primarily mediated by hemocytes [2]. Specifically, encapsulation is a major defense strategy against large foreign invading objects such as parasitoid eggs [3]. Following invasion by a large object or a parasitoid egg, the hemocytes undergo morphological changes and progressively overlap to form a multilayered cellular capsule around the target [4]. Although the hemocyte types involved in encapsulation differ among insect taxa, studies on Lepidoptera typically consider granulocytes and plasmatocytes as the principal effector cells mediating encapsulation [5]. Encapsulation is considered one of the most complex immune reactions in insects, which involving target recognition, hemocyte adhesion and aggregation, and a terminal melanization phase [6].

Numerous cytokines and extracellular components contribute to the regulation of hemocyte adhesion and aggregation of foreign objects. In model insects, such as *Drosophila*, various adhesion-related molecules modulate hemocyte aggregation and encapsulation, including extracellular matrix (ECM) proteins such as peroxinectin and laminin [7,8]. Peroxinectin (Pxt) binds to the cell surface and mediates hemocyte adhesion, thereby promoting encapsulation [9,10]. In *Bombyx mori*, a peroxinectin-like protein (BmPxtA) regulates hemocyte spreading and nodulation through prostaglandin (PG) mediated signaling within the eicosanoid pathway [11]. In *Spodoptera exigua*, eicosanoids also function as adhesion-related signals that promote hemocyte aggregation and melanization [12]. However, many of these factors are conserved ECM components or integrin-associated pathways. Studies on *M. separata* and its specific or novel adhesion mediators remain relatively limited.

During encapsulation, hemocyte–hemocyte adhesion and multilayer capsule formation are critical steps. In *Pseudoplusia includens*, encapsulation is characterized as a three-step process. Granulocytes are the first cells to attach to the target surface and are involved in the initial aggregation. Subsequently, numerous plasmatocytes are recruited and adhere to the growing capsule, forming a multilayer. Finally, granulocytes form an outer layer surrounding the capsule, marking the transition to the terminal stage of capsule formation [5]. In contrast, in *Manduca sexta*, plasmatocytes are the initial hemocyte type that adheres to the target, followed by additional cellular layers comprising both granulocytes and plasmatocytes [13]. However, the structure of the capsule and cellular layer formation patterns in *M. separata* remain unclear. During encapsulation, granulocytes release various effector molecules that coordinate hemocyte communication and capsule assembly [14]. Plasmatocyte-spreading peptide 1 (PSP1) is an ENF family cytokine expressed in the fat body and hemocytes of *P. includens* [15]. Granulocytes release factors, including PSP, to activate plasmatocytes and promote capsule formation [14]. Plasmatocytes also play important roles in the subsequent encapsulation stabilization process and melanization [6,16].

Several cellular immune-related factors have been reported in *M. separata*, including phenotypic features and cellular localization associated with encapsulation and melanization [17], the encapsulation-promoting lectin EPL [16], and recent studies of Transglutaminase (TGase) and integrins [18,19]. However, a systematic understanding of encapsulation remains lacking, as current studies mainly show that certain factors regulate hemocytes, while the specific factors produced by granulocytes and plasmatocytes and their roles in encapsulation formation are still unclear. In addition, a systematic understanding of these inter-hemocyte signaling pathways remains lacking. Therefore, the identification of novel regulators exhibiting distinct “source cell–target cell” relationships will contribute to establishing a molecular framework for hemocyte–hemocyte communication during encapsulation.

In this study, we focused on a candidate gene identified from the *M. separata* transcriptome that was significantly upregulated in capsule samples and adherent hemocytes [20]. Based on this, we further examined its expression characteristics and functional roles during encapsulation to identify a novel adhesion regulatory factor and its potential mechanism in *M. separata* encapsulation.

## 2. Materials and Methods

### 2.1. Insects

*Mythimna separata* was reared in a controlled environment insectary (23 ± 3 °C; 40–60% relative humidity; photoperiod 16 h light:8 h dark). The larvae were fed an artificial diet (Silkmate; Nihon Nosan Kogyo, Yokohama, Japan). Unless otherwise stated, all experiments were conducted using sixth-instar larvae.

### 2.2. Transcriptome Analysis

Total RNA was extracted from in vivo encapsulation samples prepared as described previously [18], and the primer sequences are listed in Table 1. First-strand cDNA was synthesized from 100 ng of total RNA with Oligo (dt)_18_ using SuperScript IV Reverse Transcriptase (Invitrogen, Thermo Fisher Scientific, Waltham, MA, USA) according to the manufacturer’s instructions. Using cDNA from in vivo encapsulation samples as a template and the primers listed in Table 1, the complete *MysERG1* coding sequence was amplified by PCR and verified on an ABI 3130 Genetic Analyzer (Applied Biosystems, Foster City, CA, USA).

Amino acid sequences corresponding to candidate genes were retrieved from the DNA Data Bank of Japan (DDBJ; http://www.ddbj.nig.ac.jp/searches-j.html, accessed on 11 November 2025) based on functional annotations reported by Yokoi et al. [20]. Homologous sequences were identified using BLASTX program on the NCBI web server; https://blast.ncbi.nlm.nih.gov/Blast.cgi, accessed on 24 February 2026). Signal peptides were predicted with SignalP 6.0 (https://services.healthtech.dtu.dk/services/SignalP-6.0/, accessed on 15 January 2026), and theoretical molecular mass and pI were estimated using ExPASy Compute pI/Mw (https://web.expasy.org/compute_pi/, accessed on 9 December 2025). Protein sequences were aligned using Clustal Omega (EBI; https://www.ebi.ac.uk/jdispatcher/msa/clustalo, accessed on 26 January 2026).

### 2.3. Gene Expression Analysis

#### 2.3.1. Sample Preparation

Eight sample types were prepared to examine *MysERG1* expression: fat body, epidermis, midgut, Malpighian tubules, circulating hemocytes, adherent hemocytes, and hemocytes engaging capsules in vivo and in vitro.

**Tissue samples (fat body, epidermis, midgut, and Malpighian tubules):** Ice-anesthetized sixth-instar larvae were dissected, and the fat body, epidermis, midgut, and Malpighian tubules were collected in separate tubes. Each tissue sample was washed thrice in phosphate-buffered saline (PBS) to remove contaminating materials and ensure that no other tissues were mixed.

**Circulating hemocytes:** Hemolymph was collected into pre-chilled tubes from multiple sixth-instar larvae, with 20 µL extracted from each individual. Samples from five larvae were pooled to generate a stock solution for subsequent use.

**Adherent hemocytes:** Following the same bleeding procedure, 160 µL IPL-41 insect medium (Thermo Fisher Scientific, Waltham, MA, USA) and 20 µL 10 mM diethyldithiocarbamate (DDC) were added to 20 µL hemolymph. The suspension was spread on grass slides and incubated at 27 °C for 1.5 h. The supernatant was removed, the slides were washed twice with PBS, and adherent cells were recovered with a cell scraper. Adherent granulocyte and plasmatocyte samples, isolated as described above, were prepared similarly.

**Hemocytes engaging capsules in vitro:** Polystyrene beads (φ 45 µm; Polysciences, Inc., Warrington, PA, USA) were washed with PBS and resuspended in 20 µL PBS at approximately 200 beads/µL. For each 20 µL hemolymph sample (from the hemocyte stock solution), 160 µL IPL-41 and 20 µL 10 mM DDC were added, followed by 1 µL of bead suspension. Tubes were incubated in a hybridization incubator with rotary shaking (Multi-Shaker Oven HB; TAITEC, Koshigaya, Japan) at 27 °C for 2 h, and subsequently centrifuged to remove the medium and washed twice with PBS.

**Hemocytes engaging capsules in vivo:** Ice-anesthetized, surface-sterilized sixth-instar larvae received three φ 600 µm polystyrene beads (Polysciences, Inc.) through an incision at the second abdominal proleg. The site was then ligated using a nylon thread (Figure S1). After 15 min of incubation, the larvae were dissected, and the beads were recovered.

**Isolation of granulocytes and plasmatocytes:** Larvae were ice-anesthetized and surface-sterilized, and the hemolymph was collected from an incision in the second prothoracic leg. Granulocytes and plasmatocytes were separated as previously described [21]. Briefly, fresh hemolymph was mixed gently with anticoagulant II (0.098 M NaOH, 0.146 M NaCl, 0.017 M EDTA) and adjusted to a final pH of 4.5 using citric acid at a ratio of 20 µL hemolymph to 100 µL anticoagulant II. Discontinuous Percoll cushions (100 µL each at 35%, 40%, 45%, 55%, and 70%) were prepared by diluting Percoll with anticoagulant I (0.613 M NaOH, 1.563 M NaCl, 0.163 M EDTA, and 0.103 M citric acid). at appropriate ratios and carefully layered in 1.5 mL tubes. The gradients were pre-centrifuged at 1200× *g* for 20 min at 4 °C. The hemolymph mixtures were then layered on top and centrifuged at 700× *g* for 15 min. The plasmatocyte and granulocyte fractions were collected from above the first and second layers, respectively. For subsequent use, cells were washed to remove anticoagulant: pellets were resuspended in 10 volumes of 0.21 M NaCl, then 200 µL of Percoll adjusted to be isosmotic with NaCl was injected beneath the NaCl layer to create a sharp interface. Samples were centrifuged at 200× *g* for 5 min at 4 °C to concentrate hemocytes at the interface (hemocytes are shown on different interfaces in Figure S2), which was then carefully collected and resuspended in IPL-41 insect medium.

For morphological identification, each hemocyte fraction was smeared onto glass slides and incubated at 27 °C for 30 min. Granulocytes were stained with neutral red (Sigma-Aldrich, St. Louis, MO, USA) (50 µL of 0.1 mg/mL) for 5 min [22]. After three washes with PBS (3 min each), the cells were examined under a fluorescence microscope (Leica Microsystems, Wetzlar, Germany). Cell types were assigned based on morphology [17] and neutral red fluorescence, and their proportions were quantified.

#### 2.3.2. Reverse Transcription Quantitative PCR (RT-qPCR)

Gene expression was quantified by RT-qPCR using Luna Universal qPCR Master Mix (New England Biolabs, Ipswich, MA, USA) on a Thermal Cycler Dice Real-Time System (Takara Bio, Kusatsu, Japan). Reactions (10 µL) were performed according to the manufacturer’s instructions using the primers listed in Table 1. The cycling condition was as follows: 94 °C for 30 s, 40 cycles of 94 °C for 15 s and 60 °C for 30 s, followed by 72 °C for 10 min. Ribosomal protein RpL32 (accession number AB669190) served as an internal reference. The relative expression level of *MysERG1* was calculated using the 2^−ΔΔCt^ method [23].

### 2.4. Expression and Purification of Recombinant Proteins

The MysERG1 coding sequence without the signal peptide was amplified by PCR using primers containing *Bam*HI and *Not* I restriction sites (Table 1). The PCR product and pGEX-4T-1 (Cytiva, Marlborough, MA, USA) were double-digested with *Bam*H I/*Not* I and ligated to generate a GST-tagged MysERG1 expression construct. The construct was introduced into *Escherichia coli* BL21 (DE3) competent cells. Recombinant protein expression was induced with 0.1 mM IPTG at 16 °C overnight, producing GST-tagged MysERG1. Cells were harvested and lysed using the xTractor Buffer (Takara Bio Inc., Kusatsu, Shiga, Japan), and GST fusion proteins were purified using Glutathione Sepharose beads (Cytiva, Marlborough, MA, USA) following the manufacturer’s protocol. The proteins were then eluted in elution buffer (50 mmol/L Tris-HCl, 10 mmol/L glutathione, pH 8.0). As a control, GST protein was purified from BL21 (DE3) cells transformed with intact pGEX-4T-1 using the same procedure. Protein concentrations were determined using a Qubit Protein Assay Kit (Invitrogen, Thermo Fisher Scientific, Waltham, MA, USA).

For SDS–PAGE and Western blotting, 2 µL of each purified protein was separated by 7% SDS–PAGE and transferred to Immobilon-P PVDF membranes (Merck Millipore, Burlington, MA, USA). Immunoblotting was performed following the method described previously [19]. Briefly, membranes were blocked with Dig Wash and Block Buffer (Roche Diagnostics GmbH, Mannheim, Germany) for 30 min at 26 °C, then incubated with rabbit anti-GST-tag primary antibody (1:10,000; Cosmo Bio Co., Ltd., Tokyo, Japan) overnight at 4 °C. After washing in PBST (prepared using phosphate-buffered saline with Tween 20 (PBS-T) tablets, pH 7.4; Takara Bio Inc., Kusatsu, Shiga, Japan), the membranes were incubated with anti-rabbit IgG (Fc)–AP conjugate (1:10,000; Promega, Madison, WI, USA) for 30 min at 26 °C. Following additional washes, membranes were developed with CDP-Star chemiluminescent substrate (Roche Diagnostics, Basel, Switzerland), and the signals were captured on a chemiluminescence film (Cytiva, Marlborough, MA, USA). Representative SDS–PAGE and Western blot images of the purified proteins are shown in Figure S3.

### 2.5. Immunofluorescence Detection

Pre-chilled tubes were prepared with 130 µL IPL-41 and 20 µL 10 mM DDC, and then 40 µL rMysERG1 or GST (150 ng/µL) was added onto glass slide. Hemolymph (10 µL) from sixth-instar larvae was mixed and incubated at 27 °C for 1.5 h. After removing the supernatant and washing twice with PBS, cells were fixed in 4% paraformaldehyde (100 µL) at 4 °C for 15 min, washed twice in PBS (3 min each), permeabilized with 0.3% Triton X-100 at 27 °C for 10 min, and washed twice with PBS. A blocking solution (Dig Wash and Block Buffer; Roche Diagnostics GmbH) was applied at 27 °C for 40 min. The samples were incubated with rabbit anti-GST tag primary antibody (1:10,000; Cosmo Bio) at 27 °C for 30 min, washed three times with PBST, and incubated with Alexa Fluor 546 goat anti-rabbit IgG (1:2000; Thermo Fisher Scientific) at 27 °C for 30 min. After three washes with PBST, the images were captured using a fluorescence microscope.

### 2.6. Hemocyte Adhesion Assay

Pre-chilled tubes were preloaded with 130 µL IPL-41 and 20 µL 10 mM DDC. rMysERG1 and rGST were diluted in 1× PBS to 150 ng/µL; 40 µL of each protein was added to separate tubes. Hemolymph (10 µL) from sixth-instar larvae was added, mixed, and diluted with IPL-41 to 5 × 10^3^ cells/µL. Aliquots (10 µL) were loaded into a hemocytometer. After a 5 min incubation at 27 °C, the chambers were washed twice with PBS, and the supernatant was transferred to a new hemocytometer. Then, 200 µL IPL-41 was added after washing, and samples were incubated at 27 °C for 30 min to allow all hemocytes in the supernatant to adhere to the new hemocytometer for counting. Granulocytes and plasmatocytes adhering to the chamber surface were counted under a fluorescence microscope (Leica). Each treatment consisted of five biological replicates.

### 2.7. Hemocyte Aggregation Assay

Polystyrene beads (φ 45 µm) were incubated overnight at 4 °C with rMysERG1, GST (each 150 ng/µL). Pre-chilled tubes were prepared with 160 µL IPL-41 and 20 µL 10 mM DDC; 20 µL hemolymph (including all types of hemocytes) from sixth-instar larvae was added, and the mixture was gently transferred to 6-well chambered slides (SPL Life Sciences, Pocheon-si, Republic of Korea). For each well, approximately 200 pretreated beads (1 µL) were added. The samples were agitated in a rotator at 27 °C for 2 h (five biological replicates per treatment). Beads exhibiting visible hemocyte coats were scored under a Leica microscope; beads whose apparent diameter exceeded 90 µm owing to hemocyte wrapping were defined as “aggregated.”

### 2.8. Double-Stranded RNA Synthesis and RNA Interference (RNAi)

To synthesize double-stranded RNA (dsRNA), a 270-bp *MysERG1* cDNA fragment (primers in Table 1) was amplified using cDNA from in vivo encapsulation samples (Section 2.3.1) as a template and cloned into the pMD20 T-vector. The insert was verified by Sanger sequencing using an ABI 3130 Genetic Analyzer. The T7 promoter was appended by PCR using T7-MCS primers (Table 1), and dsRNA was synthesized using the MEGAscript RNAi Kit (Thermo Fisher Scientific). As a negative control, the green fluorescent protein (GFP) gene sequence was derived from the pEGFP plasmid (Clontech, Mountain View, CA, USA), and dsEGFP was synthesized using the procedure described above. dsRNAs were diluted in kit elution buffer to 500 ng/µL and stored at −20 °C.

For injections, 10 µL dsMysERG1 or dsEGFP was administered into ice-anesthetized sixth-instar larvae using a glass capillary and a CellTram microinjector (Eppendorf, Hamburg, Germany). Larvae were maintained at 23 ± 3 °C. After 48 h, hemocytes were collected, and *MysERG1* knockdown was evaluated using RT-qPCR.

### 2.9. In Vitro Encapsulation Assay

Following ice anesthesia and surface sterilization, 20 µL hemolymph from each dsRNA-treated larva was mixed with 160 µL IPL-41 and 20 µL 10 mM DDC. One φ 600 µm polystyrene bead was added per tube, and samples were incubated in a rotating incubator at 27 °C for 3 h. Beads were recovered and washed twice with PBS; each group contained 10 beads. Each bead was imaged individually under a fluorescence microscope (Leica). The capsule cross-sectional area and mean pixel optical density (OD) were quantified using the ImageJ version 15.4g [24]. For each image, the capsule Region of interest (ROI) was delineated, and a nearby acellular region was selected as the background ROI. Mean pixel OD was calculated as OD = −log_10__(Mean_sample/Mean_bg), where Mean_sample and Mean_bg are the mean gray values of the sample and background ROIs, respectively.

### 2.10. Statistical Analysis

All experiments were performed at least three times, with similar results. Graphs were prepared using Prism 10 (GraphPad Software, San Diego, CA, USA), and statistical analyses were conducted using SPSS version 29.0.1(SPSS Inc., Chicago, IL, USA). Data are presented as mean ± standard error of the mean (mean ± SEM). Differences between the two groups were assessed using two-tailed Student’s *t*-tests; multiple comparisons were analyzed using one-way ANOVA followed by Tukey’s post hoc test. Differences were considered statistically significant at *p* < 0.05.

## 3. Results

### 3.1. Identification of MysERG1

Based on the *M. separata* transcriptome database [20], we identified a novel gene that increased expression levels in hemocytes engaging capsules, and named it *Mythimna separata Encapsulation-Related Gene 1* (*MysERG1*; DDBJ accession number LC901172). It encoded a 121 aa protein (excluding the stop codon). ExPASy analysis predicted a theoretical molecular weight of 11.37 kDa and an isoelectric point (pI) of 8.70. Amino acid composition analysis revealed that the protein was rich in Ser/Thr/Pro (22.3%). MysERG1 contains a clear hydrophobic region at the N-terminus, consistent with a typical signal peptide. SignalP 6 prediction further indicated a high probability of an N-terminal signal peptide (type: Sec/SPI), with the most likely cleavage site located immediately after the hydrophobic region (predicted cleavage between residues 16 and 17; probability = 0.983394).

**Figure 1:**
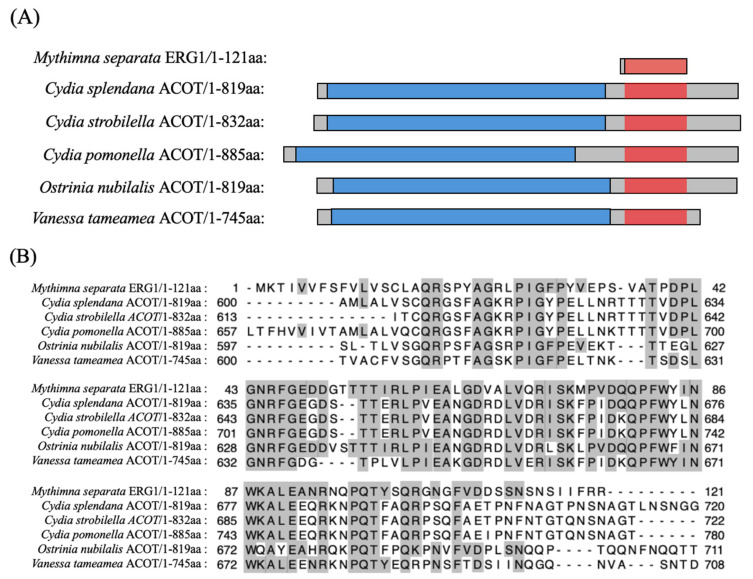
Sequence comparison of *Mythimna separata* ERG1 and lepidopteran acyl-CoA hydrolase (ACOT). (**A**) Schematic representation of full-length ACOT proteins and MysERG1. Gray bars indicate the full-length proteins. The predicted carboxylesterase family domain is shown in blue. The region exhibiting sequence similarity to MysERG1 is highlighted in red. (**B**) Multiple sequence alignment of MysERG1 with lepidopteran ACOT homologs. Residues in ACOT homologs that are identical to the corresponding MysERG1 residues are shaded in gray. Dashes indicate alignment gaps. Numbers indicate amino-acid positions in each sequence. aa, amino acids.

A comparison of the full-length level revealed significant difference among the putative homologs in lepidopteran insects (Figure 1A). MysERG1 did not show homology to known proteins (Table S1); however, it was partially similar to acyl-CoA hydrolases (ACOTs) from many lepidopteran insects (Figure 1B); *MysERG1* encoded a short protein (121 aa), whereas the ACOT homologs were substantially longer (approximately 745–885 aa) and contained a predicted carboxylesterase family domain that was absent in MysERG1. These results indicate that MysERG1 shares short amino acid regions of similarity with ACOT proteins; however, its phylogenetic placement remains unclear. Therefore, given the substantial differences in full-length sequence from known ACOTs, MysERG1 was considered a putative novel gene in this study.

### 3.2. MysERG1 Was Expressed in Adherent Hemocytes

**Figure 2:**
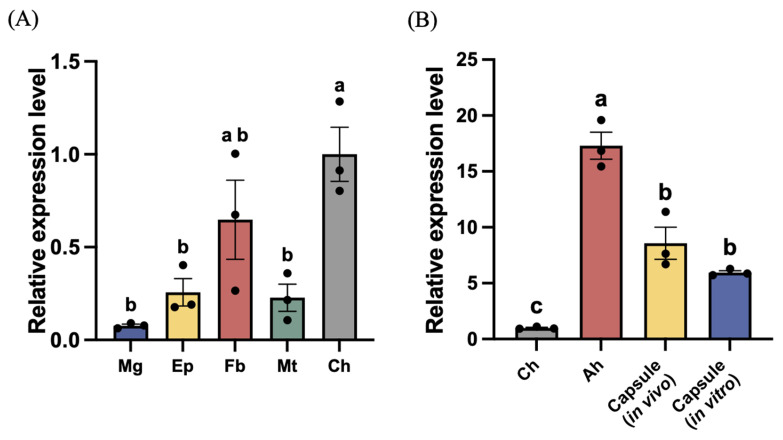
Relative expression level of *MysERG1*. (**A**) *MysERG1* expression levels in the midgut (Mg), epidermis (Ep), fat body (Fb), Malpighian tubules (Mt), and circulating hemocytes (Ch). Data were analyzed using one-way ANOVA (*F*(4,10) = 9.15, *p* = 0.0022). Error bars show means ± SEM (n = 3 biological replicates). Letters indicate statistically significant differences (Tukey’s multiple comparisons test, *p* < 0.05). (**B**) *MysERG1* expression levels in circulating hemocytes (Ch), adherent hemocytes (Ah), and two encapsulation samples. Data was analyzed using one-way ANOVA (*F*(3,8) = 52.34, *p* < 0.001). Error bars show means ± SEM (n = 3). Letters indicate statistically significant differences (Tukey’s multiple comparisons test, *p* < 0.05).

We compared *MysERG1* transcript levels in five tissues (Figure 2A). *MysERG1* showed the highest expression in circulating hemocytes, whereas its expression in the midgut, epidermis, fat body, and Malpighian tubules was comparatively low (*p* < 0.05). Among the non-hemocyte tissues, the fat body exhibited relatively higher *MysERG1* expression than the midgut, epidermis, and Malpighian tubules, but remained lower than that in circulating hemocytes.

We then compared *MysERG1* transcript levels across four sample types constructed from hemocytes (Figure 2B). *MysERG1* expression was highest in adherent hemocytes, followed by in vivo and in vitro encapsulation samples. Expression in all three groups was significantly higher than that in circulating hemocytes (*p* < 0.05). After 1.5 h of culture on glass slides, *MysERG1* expression increased notably in adherent hemocytes, indicating that *MysERG1* may regulate hemocyte adhesion or recognition. *MysERG1* was also significantly upregulated in both in vivo and in vitro encapsulation samples, suggesting that it was involved in encapsulation.

### 3.3. MysERG1 Was Highly Expressed in Plasmatocytes

To determine which hemocyte type expressed *MysERG1*, we separated the two principal hemocyte types involved in lepidopteran encapsulation—granulocytes and plasmatocytes—using discontinuous Percoll gradients. Using this method, 93% granulocytes and 82% plasmatocytes were recovered from *M. sexta* [21]. Owing to their distinct buoyant densities, the two cell types were separated into different layers. After separation, the cells were allowed to adhere and spread on glass slides for 30 min. Granulocytes were stained with neutral red, and the separation efficiency was evaluated by combining the red fluorescence signal with the identified morphology.

**Figure 3:**
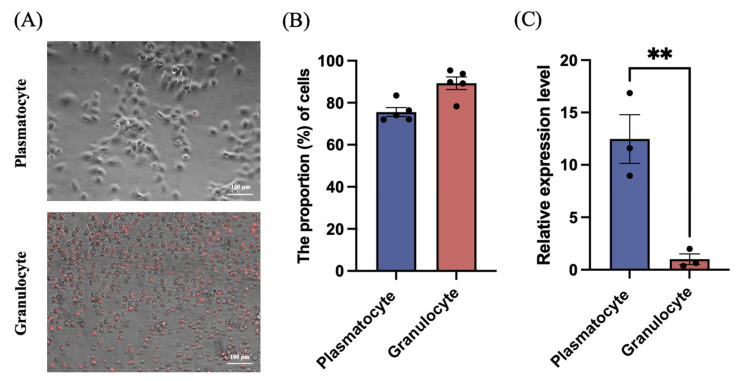
Gene expression of *MysERG1* in different hemocyte types. (**A**) Representative merged images (fluorescence overlaid on bright-field) of adherent plasmatocytes (**top**) and granulocytes (**bottom**). Scale bars, 100 μm. (**B**) Proportion (%) of cells after separation showing plasmatocytes and granulocytes (n = 5 biological replicates). (**C**) Relative *MysERG1* expression in adherent plasmatocytes and granulocytes measured by reverse transcription quantitative PCR. Data are expressed as mean ± SEM (n = 3 biological replicates). Asterisks (**) indicate significant differences (Student’s *t*-test, *p* = 0.0085 < 0.01, *df* = 4).

Representative images of the purified fractions are shown in Figure 3A. After incubation, the plasmatocytes exhibited pronounced spreading, producing a larger cell profile, whereas the granulocytes showed comparatively limited deformation and largely retained a spherical profile with clear neutral red fluorescence. Quantification indicated post-separation purities of 89.31% for granulocytes and 75.58% for plasmatocytes (Figure 3B). RT-qPCR analysis revealed that MysERG1 transcript levels were significantly higher in adherent plasmatocytes than in adherent granulocytes (Figure 3C).

### 3.4. rMysERG1 Bound to Granulocytes

**Figure 4:**
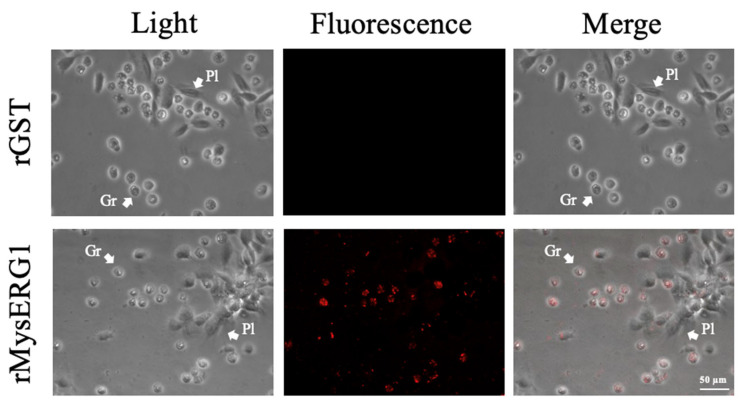
Localization of rMysERG1 in hemocytes. Hemocytes were incubated with rMysERG1 or rGST (control). Binding of recombinant proteins to the hemocytes was detected using a rabbit anti-GSTtag primary antibody, followed by an Alexa Fluor 546-conjugated goat anti-rabbit IgG secondary antibody. Hemocytes were observed under light and fluorescence microscopy; rMysERG1-derived signals are shown in red. Gr, granulocyte; Pl, plasmatocyte. Scale bars, 50 μm.

Immunofluorescence detection using a GST-specific primary antibody showed that when hemocytes were incubated with rMysERG1, a red fluorescence signal was detected in granulocytes but not in plasmatocytes. In contrast, no red fluorescence signal was observed in hemocytes incubated with rGST (control protein) (Figure 4).

### 3.5. rMysERG1 Promoted Granulocyte Adhesion

**Figure 5:**
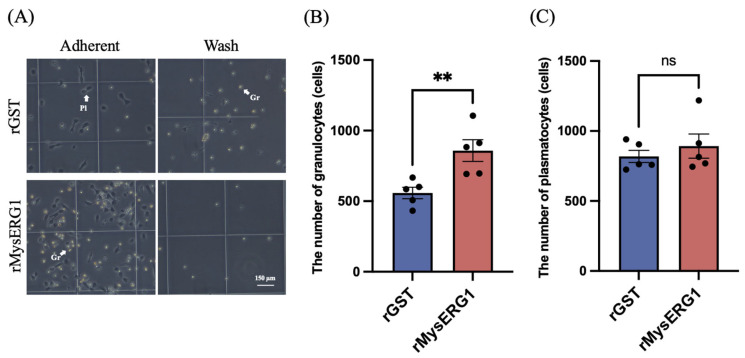
The effect of recombinant MysERG1 on hemocyte adhesion (**A**). After 15 min of incubation, images of the recovered supernatant cells (Wash) and the cells that adhered to the slide (Adherent) were captured. Gr, granulocyte; Pl, plasmatocyte. Scale bars, 150 μm. (**B**) The number of adherent granulocytes per field. Data are expressed as mean ± SEM (n = 5 biological replicates). Asterisks (**) indicate significant differences (Student’s *t*-test, *p* = 0.0087 < 0.01, *df* = 8). (**C**) The number of adherent plasmatocytes per field. Data are expressed as mean ± SEM (n = 5 biological replicates). Student’s *t*-test was used to analyze the significance of differences between groups (*p* = 0.4696 > 0.05, *df* = 8); ns, not significant.

To examine whether rMysERG1 influences hemocyte adhesion, circulating hemocytes were incubated with rMysERG1 or rGST (control protein) and allowed to settle on a hemocytometer chamber glass. As shown in Figure 5A, rMysERG1 treatment resulted in an apparent increase in the number of granulocytes adhering to the original chamber surface, whereas the number of plasmatocytes showed no significant change (Figure 5B,C). In the rMysERG1-treated group, fewer granulocytes remained suspended in the supernatant, whereas under rGST conditions, more granulocytes were observed floating in the supernatant. Overall, these results indicated that treatment with rMysERG1 promoted the rapid adhesion of granulocytes to the glass surface. Moreover, this finding supports the conclusion that rMysERG1 enhances granulocyte adhesion during early incubation stages.

### 3.6. rMysERG1 Promoted Hemocyte Aggregation

**Figure 6:**
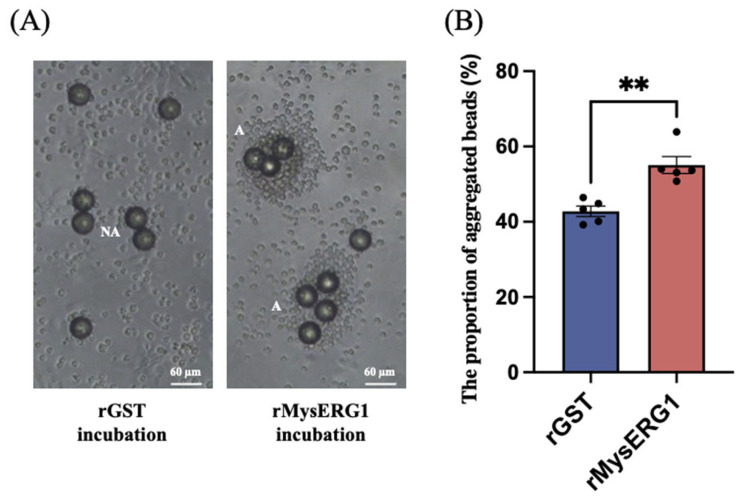
rMysERG1 promotes hemocyte aggregation on beads. (**A**) Representative bright-field images after incubation with beads pre-treated with rGST or rMysERG1; A, aggregated; NA, non-aggregated. Scale bars, 60 μm. (**B**) Analysis of the proportion of aggregated beads among the total number of beads. Beads with an apparent diameter ≥ 90 μm owing to hemocyte wrapping were defined as “aggregated”. Data are expressed as mean ± SEM (n = 5 biological replicates). Asterisks (**) indicate significant differences (Student’s *t*-test, *p* = 0.0017 < 0.01, *df* = 8).

Polystyrene beads were pre-incubated with rMysERG1, mixed with hemolymph, and incubated for 2 h before scoring aggregation. Only aggregates whose apparent diameter exceeded twice that of a single bead (≥90 µm) were counted. As shown in Figure 6A, beads coated with rGST exhibited limited hemocyte deposition, consistent with early encapsulation, whereas rMysERG1-coated beads were frequently surrounded by dense hemocyte masses. In certain cases, multiple beads were bridged together by aggregated hemocytes. Quantitatively, the proportion of aggregated beads was significantly higher for rMysERG1-coated beads than for the rGST controls, indicating that rMysERG1 enhanced hemocyte aggregation.

### 3.7. MysERG1 Knockdown Suppressed the Encapsulation Process

**Figure 7:**
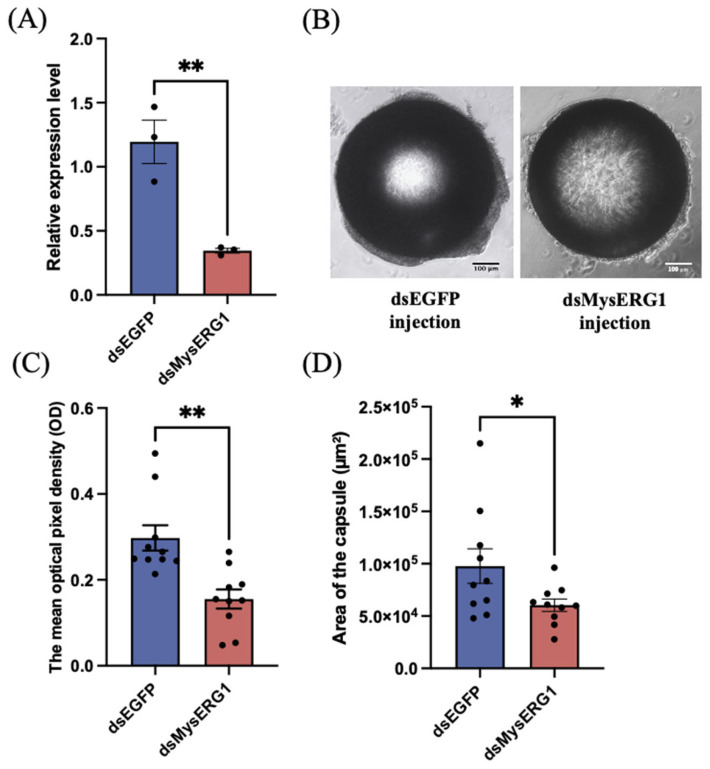
The effect of *MysERG1* knockdown on the encapsulation response. (**A**) Confirmation of gene knockdown by reverse transcription quantitative PCR. Error bars show means ± SEM (n = 3 biological replicates). Asterisks (**) indicate statistically significant differences (Student’s *t*-test, *p* = 0.0075 < 0.01, df = 4). (**B**) Image of beads forming capsules. Scale bars, 100 μm. (**C**) The mean pixel optical density (OD) of the capsules. Error bars show means ± SEM (n = 10 biological replicates). Asterisks (**) indicate statistically significant differences (Student’s *t*-test, *p* = 0.0011 < 0.01, *df* = 18). (**D**) Area of hemocytes adhering to the surface of the beads (capsules). Error bars show means ± SEM (n = 10 biological replicates). Asterisks (*) indicate statistically significant differences (Student’s *t*-test, *p =* 0.0471 < 0.05, *df* = 18).

At 48 h after dsRNA injection, *MysERG1* transcript levels were significantly reduced in larvae treated with dsMysERG1 compared to those in dsEGFP controls (Figure 7A), indicating effective gene knockdown. When φ 600 µm beads induced encapsulation, the recovered capsule from dsMysERG1-treated larvae appeared less compact than those from dsEGFP-treated larvae (Figure 7B). Image-based quantification showed that the mean pixel optical density of the capsule was significantly lower after *MysERG1* knockdown, indicating lighter pigmentation and reduced local cell density. In addition, the capsule cross-sectional area notably decreased relative to that of the controls. Collectively, these results demonstrate that *MysERG1* is required for robust capsule formation and contributes to both capsule compactness and size during encapsulation.

## 4. Discussion

This study identified a new gene involved in cellular immune regulation, *MysERG1*, and analyzed its expression and function. MysERG1 contains an N-terminal signaling peptide, suggesting that it enters the secretory pathway. Although MysERG1 exhibits minimal overall homology with known immune-related proteins, it contains local amino acid regions similar to lepidopteran ACOTs. ACOTs hydrolyze acyl-CoA to release free fatty acids and contribute to maintaining lipid balance. Certain fatty acids, particularly arachidonic acid (AA), are precursors of immune-related lipid mediators [25]. In mammals, AA can be supplied by phospholipase A_2_ (PLA_2_)-mediated release from membrane phospholipids as well as through an alternative pathway: arachidonyl-CoA (AA-CoA) is hydrolyzed by ACOT to generate free AA [26]. A recent study involving *Myzus persicae* identified an upregulated ACOT family member, thioesterase superfamily member 6a (MpTHEM6a), indicating that insects may also harbor ACOT-like proteins that regulate the balance between acyl-CoA and free fatty acids, thereby affecting AA availability and downstream eicosanoid levels [27]. In insects, ACOTs are frequently enriched in lipid-active tissues such as the fat body and midgut [28,29]. The released free fatty acids, notably arachidonic acid-related precursors, can enter eicosanoid biosynthesis pathways and regulate hemocyte spreading, aggregation, nodulation, and encapsulation through signals such as prostaglandin E2 (PGE2) [30,31]. As MysERG1 lacks the carboxylesterase family domain conserved in ACOT proteins and shows a substantial difference in full-length sequence length, we currently believe that MysERG1 regulates encapsulation through a mechanism not shared by ACOTs. However, MysERG1 may function as a previously uncharacterized domain related to ACOT proteins, thereby participating in cellular immune regulation. This hypothesis requires further investigation in future studies.

*MysERG1* expression was increased in adherent hemocytes and hemocytes forming capsules. This expression pattern is similar to that reported in several studies. For example, Cox-Foster et al. reported FAD-dependent glucose dehydrogenase (GLD) in *Manduca sexta* [32]. Although its expression is barely detectable in freshly collected circulating hemocytes, it increases substantially in plasmatocytes that have spread on glass slides and in hemocytes forming capsules around latex beads. Similarly, the strong induction of *MysERG1* in adherent hemocytes may indicate that hemocytes recognize the glass slide as a large, non-self surface. The shift in hemocytes from a circulating state to an adherent and spreading state is a key initial process that induces encapsulation responses [33]. Integrin gene expression is also induced by cell adhesion and encapsulation. Levin et al. identified a hemocyte-specific integrin gene expressed in the hemocytes of *M. sexta* [34]. When hemocytes transition from circulation to form a multilayer capsule on a foreign surface, the activity of this integrin must be regulated to support the change from a non-adhesive to an adhesive state [34]. Mao and Furukawa [18] reported that, during encapsulation, hemocytes forming the capsule showed notable upregulation of several integrin genes in *M. separata*. Based on these findings, we proposed that *MysERG1* contributes to the regulation of encapsulation.

*MysERG1* transcript levels were significantly higher in adherent plasmatocytes than in adherent granulocytes. In contrast, immunofluorescence showed that the rMysERG1 signal was detected mainly in the granulocytes. This pattern of expression in one hemocyte type, along with its association with a different type, is consistent with cytokine-like regulation [35]. Similar “source cell–target cell” separation has been reported for cytokines involved in lepidopteran encapsulation. For example, *plasmatocyte-spreading peptide* (*PSP1*) in *P. includens* is expressed in hemocytes and the fat body, and promotes plasmatocyte recruitment and spreading [14], thereby supporting multilayer capsule formation. The *B. mori* paralytic peptide (BmPP), an ortholog of *P. includens* PSP [36], is mainly expressed in the fat body [37] and stimulates plasmatocyte spreading [38]. The ENF peptide family of proteins does not act only within producing cells; rather, they function as signals that regulate hemocyte behavior and activation [39]. MysERG1 is assumed to affect distant hemocytes to activate immune reactions. Our results indicate that MysERG1 functions in granulocytes. It is possible that a specific receptor for recognition of MysERG1 is preferentially expressed in granulocytes but not in plasmatocytes; this could explain why MysERG1 predominantly functions in granulocytes. Taking this speculation into consideration, MysERG1 may function as a novel type of cytokine that regulates the insect immune system.

rMysERG1 promotes the adhesion of granulocytes to glass slides but does not enhance plasmatocyte adhesion. This differs from PSP1 in *P. includens*, which stimulates plasmatocyte spreading and suppresses granulocyte spreading [14]. In addition, rMysERG1 showed an aggregating effect on hemocytes, resembling the activity of the *M. separata* hemocyte chemotactic peptide (HCP) [40]. Notably, the function of MysERG1 shows similarities to that of peroxinectin, a well-characterized hemocyte adhesion protein. Peroxinectin has been reported to promote hemocyte adhesion and spreading through integrin-mediated interactions [9]. Similarly, rMysERG1 enhanced hemocyte adhesion and aggregation, suggesting a comparable role in regulating hemocyte behavior. However, MysERG1 lacks known conserved domains characteristic of peroxinectin or other adhesion-related proteins, and its molecular mechanism remains unclear. These functional similarities suggest that MysERG1 may represent a distinct type of hemocyte-regulating factor, potentially acting through a novel pathway. Certainly, we cannot fully exclude the possibility of non-specific effects, such as surface coating or contaminants, and further controls will be required to confirm the specificity of MysERG1 activity.

After *MysERG1* knockdown, the capsule wall became looser, and its size was reduced. The effect of *MysERG1* knockdown was consistent with the phenotypes reported for other pro-encapsulation factors. For example, *O. furnacalis* encapsulation-promoting protein 1 (OfEPP1) promotes encapsulation mainly by mediating hemocyte aggregation rather than spreading, and knockdown of *OfEPP1* reduces the proportion of encapsulated beads [41]. Similarly, in *M. sexta*, knockdown of integrin β1 disrupts hemocyte adhesion and weakens the ability to encapsulate beads [34]. Hu et al. quantified the extent of encapsulation by classifying capsule thickness into 13 grades and showed that knockdown of *Helicoverpa armigera* defense protein 1 (Ha-DFP1) significantly reduced capsule thickness [33]. Consistently, RNAi knockdown of *MysERG1* produced encapsulation phenotypes that corresponded with the observed cellular effects, further supporting the conclusion that MysERG1 is a potential factor that regulates hemocyte behavior. However, we cannot exclude the possibility that this phenomenon is also influenced by changes in hemocyte number or subtype composition, which were not directly assessed in this study.

**Figure 8:**
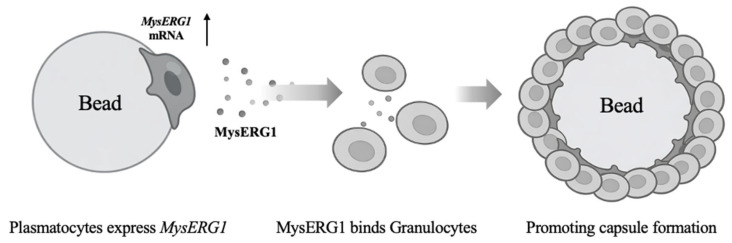
Proposed model of MysERG1 role in *Mythimna separata*. Plasmatocytes express *MysERG1*; secreted MysERG1 binds specifically to granulocytes to enhance adhesion capacity. Finally, hemocyte capsules around the foreign materials are formed by encapsulation. The arrow indicates an increase in *MysERG1* expression.

In summary, *MysERG1* expression was induced in plasmatocytes by attachment to foreign surfaces, and the released MysERG1 promoted granulocyte adhesion, thereby promoting capsule formation (Figure 8). This model suggests that the process of encapsulation in *M. separata* is similar to that in *M. sexta*, where plasmatocytes attach to the target first, and subsequent cellular layers are formed by both granulocytes and plasmatocytes [13]. Notably, during homology searches, proteins homologous to MysERG1 were identified solely as hypothetical proteins in Lepidoptera among insects, indicating that MysERG1 is not specific to *M. separata*; rather, it likely represents a universal cellular immune regulatory factor uniquely found in Lepidoptera among insects. Currently, we have not elucidated the molecular mechanisms by which MysERG1 enhances the adhesion capacity of granulocytes. Further studies, including identification of the target molecules of MysERG1, are required to elucidate the complete mechanism of the encapsulation process in lepidopteran insects.

## Figures and Tables

**Table 1:** Primers used in this study.

Primer Name	Sequence (5′ to 3′)	Purpose
*RP32*-F	TGACAAACTCAAGCGTAACTGGCG	RT-qPCR
*RP32*-R	TTGCGGAAACCATTGGGCAG	RT-qPCR
*MysERG1*-F	CTTGGTGATGTGGCTCTAGTC	RT-qPCR
*MysERG1*-R	TTGTGAGTACGTCTGTGGTTG	RT-qPCR
*MysERG1-F BamH1*	GGATCCCAAAGATCTCCATATGCAGGCAGG	rMysERG1
*MysERG1-R Not1*	GCGGCCGCTTATCTCCGGAAGATGATTGA	rMysERG1
*MysERG1*-F	AGCTGCTTGGCCCAAAGAT	dsRNA synthesis
*MysERG1*-R	TTGTGAGTACGTCTGTGGTTG	dsRNA synthesis
*T7-MCS* -F	taatacgactcactatagggTAGTCATATG	dsRNA synthesis
*T7-MCS* -R	taatacgactcactatagggCCGGGGATCC	dsRNA synthesis
*dsEGFP*-F	taatacgactcactatagggATGGTGAGCA	dsRNA synthesis
*dsEGFP*-R	taatacgactcactatagggTTACTTGTAC	dsRNA synthesis

Note: Lowercase letters represent the T7 polymerase promoter.

## Data Availability

The MysERG1 sequence generated in this study has been deposited in the DNA Data Bank of Japan (DDBJ; http://www.ddbj.nig.ac.jp/searches-j.html, accessed on 11 November 2025) under accession number LC901172. The datasets and analysis protocols used during the current study are available from the corresponding author on request.

## Supplementary Materials

The following supporting information can be downloaded at: https://www.mdpi.com/article/10.3390/insects17040372/s1. Figure S1. Larvae with the incision closed using nylon thread. (A) The second abdominal proleg of a dissected larva. Blue arrow: incision site; red arrow: 600 μm bead. (B) After inserting the bead through the incision (proleg), the wound was ligated with nylon thread. (C) The incision site ligated with nylon thread. Scale bars, 1 mm. Figure S2: Granulocytes and plasmatocytes of *Mythimna separata* separated by Percoll density gradient centrifugation. The blue arrow indicates the plasmatocyte layer, while the red arrow indicates the granulocyte layer. Figure S3: Expression of recombinant rMysERG1 and rGST proteins. (A) SDS–PAGE. Lane 1, protein molecular weight marker. Lane 2, rGST (~26 kDa); Lane 3, rMysERG1 (~38 kDa); (B) Western blotting. Lane 4, rGST (~26 kDa); Lane 5, rMysERG1 (~38 kDa); Lane 6, protein molecular weight marker. Table S1. BLAST-based homology search results for MysERG1.

## Abbreviations

The following abbreviations are used in this manuscript:

AA	Arachidonic acid
AA-CoA	Arachidonyl-CoA
ACOTs	Acyl-CoA hydrolases
BmPP	*Bombyx mori* paralytic peptide
BmPxtA	*Bombyx mori* peroxinectin-like protein A
dsRNA	Double-stranded RNA
ECM	Extracellular matrix
EPL	Encapsulation promoting lectin
GFP	Green fluorescent protein
HCP	Hemocyte chemotactic peptide
Ha-DFP1	*Helicoverpa armigera* defense protein 1
MpTHEM6a	*Myzus persicae* Thioesterase superfamily member 6a
OD	Optical density
OfEPP1	*Ostrinia furnacalis* encapsulation-promoting protein 1
PG	Prostaglandin
PGE2	Prostaglandin E2
PLA_2_	Phospholipase A_2_
PSP	Plasmatocyte spreading peptide
RNAi	RNA interference
RT-qPCR	Reverse transcription quantitative PCR
SEM	Standard Error of the Mean
Pxt	Peroxinectin
ROI	Region of interest
