# Supplementary material for: Functional Analysis of MysERG1, a Novel Immune-Related Gene in Encapsulation Regulation, in the Oriental Armyworm Mythimna separata (Lepidoptera: Noctuidae)
Source: Insects. 2026 Apr 1;17(4):372. doi: 10.3390/insects17040372 (PMC13116936; doi:10.3390/insects17040372)
Supplement: Supplementary file 1 [file insects-17-00372-s001.zip › Supplementary Material S1.pdf]

Supplementary Material

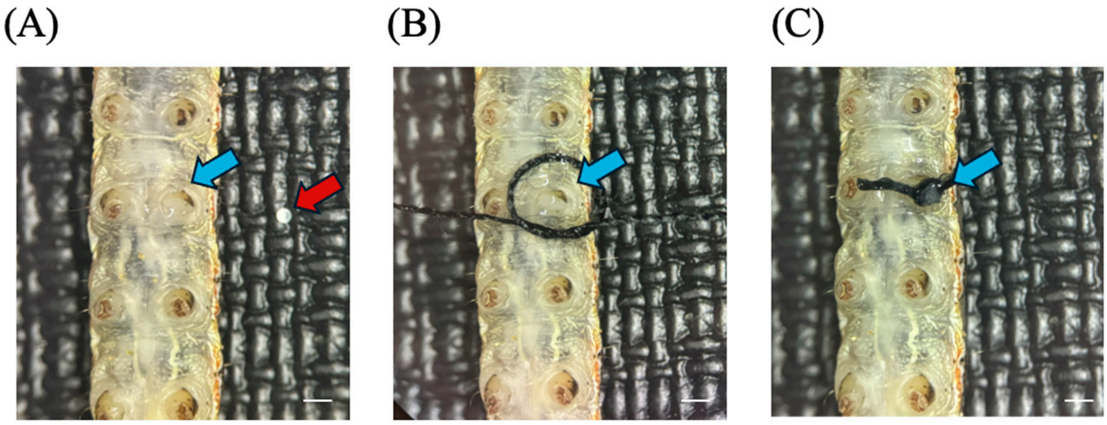

Figure S1. Larvae with the incision closed using nylon thread.(A) The second abdominal proleg of a dissected larva. Blue arrow: incision site; red arrow: 600 μm bead. (B) After inserting the bead through the incision (proleg), the wound was ligated with nylon thread. (C) The incision site ligated with nylon thread. Scale bars, 1 mm.
