# Supplementary material for: Functional Analysis of MysERG1, a Novel Immune-Related Gene in Encapsulation Regulation, in the Oriental Armyworm Mythimna separata (Lepidoptera: Noctuidae)
Source: Insects. 2026 Apr 1;17(4):372. doi: 10.3390/insects17040372 (PMC13116936; doi:10.3390/insects17040372)
Supplement: Supplementary file 1 [file insects-17-00372-s001.zip › Supplementary_Material S2.pdf]

Supplementary Material

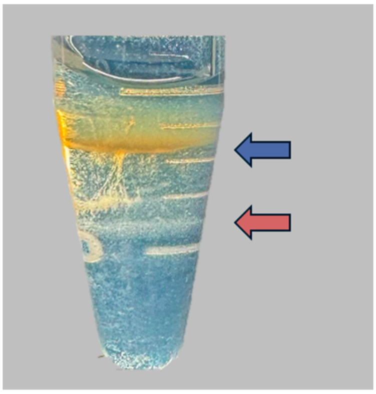

Figure S2. Granulocytes and plasmatocytes of *Mythimna separata* separated by Percoll density gradient centrifugation. The blue arrow indicates the plasmatocyte layer, while the red arrow indicates the granulocyte layer.
