# Supplementary material for: Functional Analysis of MysERG1, a Novel Immune-Related Gene in Encapsulation Regulation, in the Oriental Armyworm Mythimna separata (Lepidoptera: Noctuidae)
Source: Insects. 2026 Apr 1;17(4):372. doi: 10.3390/insects17040372 (PMC13116936; doi:10.3390/insects17040372)
Supplement: Supplementary file 1 [file insects-17-00372-s001.zip › Supplementary_Material S3.pdf]

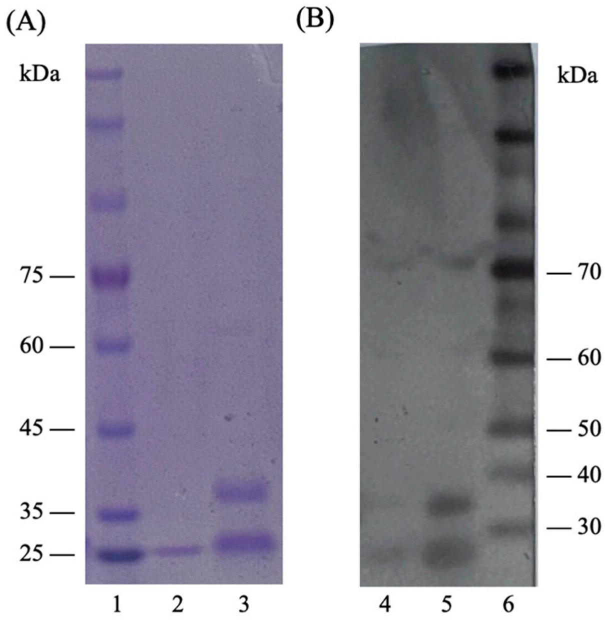

Figure S3. Expression of recombinant rMysERG1 and rGST proteins. (A) SDS–PAGE. Lane 1, protein molecular weight marker. Lane 2, rGST (~26 kDa); Lane 3, rMysERG1 (~38 kDa); (B) Western blotting. Lane 4, rGST (~26 kDa); Lane 5, rMysERG1 (~38 kDa); Lane 6, protein molecular weight marker.
